# Validation and Measurement of Attitudes Towards Gambling: The Case of the Basque Country

**DOI:** 10.1007/s10899-025-10431-w

**Published:** 2025-10-23

**Authors:** Aidée Baranda Ortiz, Iraide Fernández Aragón, Jonatan García Rabadán

**Affiliations:** Department of Sociology and Social Work, University of the Basque Country, Barrio Sarriena s/n, Leioa, 48940 Spain

**Keywords:** Attitudes, Gambling, Public opinion, basque country

## Abstract

Gambling is deeply rooted in Basque society due to its high participation. However, in recent times it has been the focus of public debates. This study aims to offer a first sociological approach to the phenomenon of gambling in the Basque Country and to explore social attitudes towards it. To this end, an attempt was made to apply the Attitudes Towards Gambling Studies, carried out in other contexts, although the results were not fully satisfactory. In response, the Synthetic Index of Attitudes towards Gambling (SIAG) was designed and applied in order to better capture the specificities of the Basque case. The SIAG has made it possible to detect a marked polarization in attitudes towards gambling. Based on the responses to different items related to regulation, danger, economic impact and (i)responsibility associated with gambling, three clearly differentiated profiles have been identified: people opposed to gambling, people who are ambivalent and people who are in favor of gambling. In summary, despite a generalized negative perception of gambling and pejorative attitudes derived from its consequences, these are not strong enough to discourage gambling or eliminate its recognition as a relevant economic agent.

## Introduction

Private gambling was decriminalised in Spain at the end of the 1970 s (Pino, 2014). It had been prohibited during the Franco dictatorship, while the State-run forms of gambling (the lottery, football pools, etc.) continued to operate. This situation ended after the restoration of democracy. The public became more sensitive to problem gambling, its accessibility and the increasing visibility of gambling establishments and advertising (Delffabbro & King, 2020). This new perspective took time to be adopted in Spain and the Basque Country. The latter case is presented here as a specific and separate situation. 

On the one hand, the Basque Government was a pioneer in the regulation of gambling (Cases, 2011). On the other hand, as a consequence of this early regulation, gambling and betting are widespread in Basque society, with over 85% of the population participating (García Rabadán et al., 2024). As a result of this new social scenario, the Basque Government created the first regional observatory on gambling in 2019^1^. Despite its widespread availability and accessibility (Villar Lama et al., 2021), gambling continues to carry a strong social stigma in the Basque Country. Rooted in a cultural tradition influenced by Catholic moral values (Pérez-Agote, 2006), gambling is often associated with vice and irresponsibility (Morales-Jiménez, 2021)—especially among older generations. Data from the Basque Gambling Observatory (Gobierno Vasco, 2017, 2022) show that public attitudes in Euskadi are notably more negative than in other Spanish regions, a factor that shapes how individuals express their views in survey contexts. 

The aim of this paper is therefore to address the perception of Basque citizens regarding gambling, the factors that influence their attitudes, and how those perceptions may vary according to different socio-demographic variables. In specific terms, we propose the application of the Attitudes Towards Gambling Scale (ATGS) and the creation of an alternative measure, given the limited results that this scale yields for the Basque case: the Synthetic Index of Attitudes Towards Gambling (SIAG). This understanding is fundamental in order to address the issue scientifically, with a view to enabling the development of effective public policies and prevention programmes that promote responsible gambling and mitigate the risks associated with problem gambling. In addition, this study will contribute to a better understanding of the phenomenon of gambling in the Basque Country, and will provide valuable information for authorities and organisations working to regulate and promote responsible gambling from a sociological perspective.

While the ATGS-8 scale is a widely used instrument for measuring gambling attitudes, its application in this study required a methodological adaptation. Given the strong social disapproval of gambling in the Basque Country—documented in previous studies and reinforced by cultural and institutional factors—the use of a standard 5-point Likert scale risked inflating the neutral midpoint due to social desirability bias. To address this, the response format was adapted to a 4-point forced-choice scale, aimed at encouraging more defined positioning. This decision, although it limits direct comparability with international studies, enhances internal validity and contextual relevance. This methodological nuance is particularly relevant in a region where gambling remains a socially sensitive and morally loaded issue.

## Theoretical Framework

From a theoretical perspective, the social profile of gambling has been normalised in recent years, as it has acquired a new public image (Smith, 2020) in the international context. In a scenario of a risk society, in which attempts are made to minimise the dangers caused by modernisation (Beck, 2002), gambling has been successfully commodified and sold to individual consumers (Young, 2010). This risk has been accepted in recent or late modernity, as Giddens (1995) calls it, due to the identity crisis that the individual suffers, and which leads to a constant calculation of future possibilities. According to Reith (2002), risk and uncertainty become the characteristics of gambling culture. Gamblers paradoxically seek meaning and order in chance. This excitement when experiencing risk is due to a search for an escape from the monotony of everyday life (Reith, 2002), and provides the individual with an opportunity to show courage and integrity in the face of risk, and thereby gain the respect of their peer group.

However, despite being considered a leisure activity, gambling has major impacts on different personal and social spheres (Latvala et al., 2019). Society’s perception of gambling and betting is crucial for guiding the normalisation of gambling and betting and therefore for identifying the consequences that can arise from the activity itself. Furthermore, its influence on self-identification as a gambler is also undeniable (Lopez-Gonzalez et al., 2019).

The identification of attitudes to gambling is therefore crucial. To that end, the Attitudes Towards Gambling Scale (ATGS), which was initially developed in Great Britain (Wardle et al., 2007), is in widespread use. An 8-item alternative was subsequently created, in which half of the items are positive statements about gambling, and the other half are negative (Canale et al., 2016) to compensate for possible biases. As shown in the items in the ATGS-8 (see Table 1), the primary focus shifted, with the removal of a moralistic stance or disdain for gambling, and it was instead focused on the right to gambling, its accessibility and its impact on society. At the same time, the scale was also adapted to accommodate both gamblers and non-gamblers.

**Table 1:** The 8-item attitudes towards gambling scale (ATGS)

1.	People should have the right to gamble whenever they want.
2.	There are too many opportunities for gambling nowadays.
3.	Gambling should be discouraged.
4.	Most people who gamble do so sensibly.
5.	Gambling is dangerous for family life.
6.	On balance gambling is good for society.
7.	Gambling livens up life.
8.	It would be better if gambling was banned altogether.

Source: Canale et al. (2016)

The scale has been used and validated in multiple locations, although as expected, the results have varied depending on the country’s economic and cultural context (Kristensen et al., 2023). In most studies that have addressed the issue of citizens’ attitudes towards gambling, i.e. the mean attitude score or trend, they have generally been negative (Delfabbro & King, 2020).

The literature using the ATGS-8 and focusing on the adult population yields very interesting results from a demographic perspective. For the most part, men have more positive attitudes than women (Pallesen et al., 2020; Fiedor et al., 2019; Salonen et al., 2014, 2017; Orford et al., 2009). Although they present more favourable perspectives towards gambling, their attitudes have become more friendly or less reticent in recent years (Tessenyi, 2023). However, this standpoint is not unique, as other studies have found no significant differences between the sexes (Andrà et al., 2022), which sparks a debate on this issue. In relation to age, which is another variable that has been presented as explanatory of the phenomenon, young people tend to have more favourable attitudes towards gambling than middle-aged people (Tessenyi, 2023; Fiedor et al., 2019; Salonen et al., 2014). Other studies have also highlighted the different standpoint of older people (Pallesen et al., 2020; McAllister, 2014), which implies a social divide based on age. While this contrast between groups is evident for gender and age, they are not the only variables that have shown their effect on attitudes towards gambling. Level of education (Àndra et al., 2022; Pallesen et al., 2020; Salonen et al., 2014), employment status (Pallesen et al., 2020), income level (Pallesen et al., 2020; Salonen et al., 2014) and even frequency of attendance at religious ceremonies (McAllister, 2014) have also been associated with conflicting positions. For all these reasons, the study of gambling from a sociological perspective becomes even more important, especially at local levels. Other research has focused on attitudes towards gambling based on variables directly linked to participation. A positive attitude has been detected among those who participate in gambling (Pallesen et al., 2020) and those who gamble most often (Fiedor et al., 2019; Canale et al., 2016; McAllister, 2014), as might be expected.

However, there are other dimensions that also influence these attitudes to gambling that are not included in the ATGS scale. Advertising contributes to some extent to participation in gambling and the decision to place bets. As an example, gamblers who had set limits on their habit reported a negative influence of advertising campaigns. This was probably due to a perception that advertisements were detrimental to efforts to reduce excessive gambling (Binde & Romild, 2019). However, advertisements containing messages that this leisure activity can lead to a happier lifestyle almost certainly have a greater appeal to adolescents, as they are in a transitional period between childhood and adulthood (Derevensky et al., 2010), with everything that this entails in terms of the passage to personal maturity.

Another area of particular interest has been the regulatory sphere, as legislation has a direct effect on both the regulation of the sector and on the context. Most people tolerate gambling and the various places where it occurs. Nevertheless, proposals are often made for these premises to be located elsewhere, away from neighbourhoods and spaces frequented by children or other vulnerable groups, and the desire is expressed that these venues should not be easily accessible to these groups (Delfabbro & King, 2020). The literature also confirms that the public is in favour of strict oversight and regulation of the industry, which is consistent with what Collins et al. (2015) call a ‘restrictive model’. This lies somewhere between complete deregulation and prohibition. These views are also in line with the so-called ‘Las Vegas model’ (Schwartz, 2006), in which gambling is ‘quarantined’ or limited to specific locations away from residential neighbourhoods. From another perspective, gambling venues or betting shops lead to NIMBY (Not in My Back Yard) effects/movements, i.e. social ‘permission’ as long as it does not directly affect the individual concerned. These phenomena have been identified and extensively studied in recent decades with relation to other sectors, including landfills, prisons, juvenile reform centres, and fracking.

Finally, Delfabrro and King (2020) offer three explanations in an effort to understand the discrepancy between behaviours and attitudes towards gambling. First, they argue that negative attitudes may be influenced by the wording of commonly used measures. The second explanation refers to the nature of the gambling population. The majority of gambling takes place through government-organised lotteries, and the people buying tickets do not approve of other forms of gambling, which implies the establishment of a dichotomy between ‘acceptable’ and ‘reprehensible’ gambling. The final explanation is the public’s limited knowledge about the extent of problem gambling and its negative consequences; and incorrect assumptions about the public’s interest in gambling.

Other scales have been developed to measure opinions of gambling. However, they were rejected because they have not been widely used, because they were conducted in different social contexts, but above all, because of the number of items used. The Gambling Attitude Survey (GAS) (Kassinove, 1998) contains 16 items, while the GABS (Breen & Zuckerman, 1999) contains 35, with 23 in its reduced GABS-23 version (Bouju et al., 2014). Rousseau and Venter (2002) created a 30-item scale from two existing scales, and added new items derived from the academic literature. In the Asian context, there are also other alternatives including the 24-item scale (Wu et al., 2012), although it is difficult to transpose to these the European, Spanish or Basque context.

## The Data

In order to fulfil the objectives described above, the data were drawn from a cross-sectional population prevalence survey titled: *“Social Perception and Sssessment of Gambling in the Basque Country.”* 1,200 face-to-face individual interviews were conducted with individuals aged 18 and over between September and October 2022. The sample was selected using probability sampling and simple stratification by sex and age.

The original questionnaire consisted of 39 items divided into 5 different thematic sections. The attitudes section included a specific battery of questions linked to the aforementioned ATGS scale, in its 8-item version. These variables were used both analyses of this scientific study: ATGS and.

In this study, the ATGS-8 (Attitudes Towards Gambling Scale) was used in an adapted version, modifying the original five-point response format to a four-point scale by eliminating the neutral midpoint. This methodological decision was based on both theoretical and contextual considerations.

From a theoretical perspective, several studies have shown that the midpoint category in Likert-type scales (e.g., the value “3” in a 1-to-5 scale) can act as an escape route to avoid taking a clear stance, especially when the topic under evaluation is morally charged or socially controversial (Krosnick, 1991; Garland, 1991). This may occur for multiple reasons: low involvement with the question, lack of knowledge, or more importantly, the desire to avoid expressing a socially uncomfortable or incorrect opinion. Survey psychology literature has noted that respondents often choose the middle option not out of genuine neutrality, but to minimize the discomfort of expressing unpopular views—particularly when the cultural or media environment strongly defines what is considered acceptable (Tourangeau et al., 2000).

In the specific case of the Basque Country, this issue is especially relevant. Euskadi is a society historically shaped by Catholic traditions, with a civic culture marked by strong normative values regarding what is considered socially and morally appropriate. Within this context, gambling has traditionally been a socially stigmatized activity, particularly among older generations, and is associated with disorder, vice, or irresponsibility. Data from the Basque Gambling Observatory (Gobierno Vasco, 2012–2022) consistently show that the social image of gambling is highly negative—more so than in other Spanish regions.

Previous studies conducted by the Observatory have found a high frequency of neutral or “don’t know/no answer” responses to statements about gambling, which may reflect evasive attitudes or fear of social judgment. To reduce this social desirability bias and encourage more defined responses, the present study adopted a four-point forced-choice scale that excludes the neutral option. This technique has been successfully used in research on morally sensitive attitudes (Revilla & Saris, 2013), as it allows researchers to better capture respondents’ latent positions—even when those positions may be uncomfortable to express. The methodological literature considers this a valid strategy when the midpoint is likely to be overused by individuals who, in fact, hold a clear opinion (Garland, 1991).

To preserve the principle of respondent autonomy and allow for genuine indecision, fieldworkers were instructed not to offer a “don’t know/no answer” option nor to mention a midpoint explicitly, but to record it if the respondent spontaneously requested it. This protocol was designed to minimize automatic or socially normative responses without artificially forcing a choice when none existed. Although we acknowledge that this adaptation limits direct comparability with studies using the original ATGS-8 format, we believe it provides greater precision and internal validity in the specific sociocultural context of the Basque Country.

## Application of the ATGS-8 Scale

Against this backdrop, this article aims to test the use of the scale in a local setting. The first part of the analysis will explore each item on the ATGS scale described above, and the construction of the index. This will provide a specific perspective on each of the aspects that make up the scale, as well as a general overview. The second part is the factor analysis technique, as previously used by other researchers (Salonen et al., 2014, 2017), has been employed.

First, Table 2 shows the mean of each item on the ATGS-8 scale in the Basque Gambling Observatory study. The overall sample mean score of 16,48 (range 8–32; midpoint 20) reflects unfavourable attitudes towards gambling. More than 95% of the interviewees agreed that there are too many opportunities to gamble, and more than 85% believed that gambling should be discouraged. The statements which the Basque population agreed with to the greatest extent are ‘Most people who gamble do so sensibly’, followed by ‘Gambling livens up life’ and ‘It would be better if gambling were banned’. In overall terms, the mean scores on a scale of 1 to 4 lie between the minimum of item 5 (1.45) and the maximum of item 4 (2.66), i.e. the range between the two is just over 1.2 points.

Furthermore, and with the aim of being able to compare the results with other international studies, although an adaptation to a 4-point scale has been made, the average has been calculated on a total of 40. Thus, category 3 has been omitted, leaving values 1, 2, 4, and 5. The average, also shown in Table 4, shows a negative attitude towards gambling on the part of Basque society, with a value of 19.11. According to the review by Delfabbro and King (2020), these results are closer to those obtained in countries such as Israel (19.5) or the Czech Republic (17.8).

**Table 2:** Mean of each item and percentage of each score on the ATGS scale

Item	Mean (about 32)	Mean† (about 40)	Strongly Disagree	Disagree	Agree	Strongly Agree
1	1.83	1.98	36.1	48.9	11	4.1
2*	1.50	1.54	1	2.8	41.6	54.6
3*	1.66	1.81	1.8	13	34.9	50.3
4	2.66	3.25	10.8	29.7	42	17.4
5*	1.45	1.5	0.5	4.4	35.1	60
6	2.39	2.8	17.8	35	37.7	9.6
7	2.57	3.14	14.7	28	43.3	14
8*	2.54	3.17	11.3	51.9	16.8	20
Total	16.48	19.11				

*Items that were reversed

†items have been calculated using the numerical values 1, 2, 4, and 5, omitting 3, in order to enable comparability with other studies

>2.5 positive attitude towards gambling

Source: prepared by the authors based on the survey ‘Social perception and assessment of gambling in the Basque Country 2022’

Beyond the simple descriptive analysis, the factor analysis (Table 3) used a Varimax rotation with Kaiser standardisation. The results of the application of this technique support the use of two factors, which is consistent with the findings of the original 14-item instrument (Orford et al., 2009). However, the first component is substantially larger, accounting for 35% of the variance, while the other component accounts for only 16%. This results in a method that contrasts positively and negatively worded items, and lacks a simple sociological explanation and is therefore difficult to analyse and make sense of.

Internal consistency was evaluated using Cronbach’s alpha. The Basque version of the ATGS-8 (4-point) demonstrated an alpha of 0.719, with the item-total correlations ranging from 0.285 to 0.536. This result aligned with the Finnish version (Salonen et al., 2014) and was not very different compared with the British version, which reported and alpha of 0.76 (Wardle et al., 2011).

**Table 3:** Rotated component matrix

	Component	
	1	2
Most people who gamble do so sensibly.	0.783	
On balance gambling is good for society.	0.764	
It would be better if gambling was banned altogether.	0.695	
Gambling livens up life.	0.531	
There are too many opportunities for gambling nowadays.		0.753
Gambling should be discouraged.		0.745
Gambling is dangerous for family life.		0.691
People should have the right to gamble whenever they want.		0.553
Source: prepared by the authors using the SPSS software package		

Source: prepared by the authors

Consequently, in addition to confirming that the Basque population’s overall attitudes towards gambling in each item are quite negative, and that gamblers have more positive attitudes, the absence of significant results prevents us from progressing in the analysis of the differences in each socio-demographic group. This statistical limitation means we cannot explore the profiles within attitudes or variations in attitudes, resulting in a superficial analysis of attitudes. We therefore decided to continue working with a scale for measuring opinion on gambling, based on the ATGS scale with new items added to it to complement the general framework.

## Creating the Synthetic Index of Attitudes Towards Gambling (SIAG)

Having verified that the ATGS-8 scale does not yield significant results in the case of the Basque Country, the aim of this study is to construct a synthetic index that measures the attitude of the Basque population to its various dimensions, using the same items used by the ATGS scale.

The data used to create the index are taken from the 2022 survey, which was the first of its kind in the Basque Country. As for the methodology used to construct the index, although some authors use additive scales, it is more common to use factor analysis as a prior step before creating a synthetic or mathematical index (Poza Lara & Fernández Cornejo, 2010; Temes, 2014; Fernández-García et al., 2018; Fernández-Aragón et al., 2021). The scores of the extracted factors or dimensions were saved as variables, and their explanatory power is used to weight the index, through their levels of saturation.

Factor analysis depends almost entirely on the variables selected for the design of the model. This process ‘operates in 3 steps: the selection of dimensions, the selection of aspects of each dimension, and the selection of measures or versions of the aspects’ (Janson, 1980, p. 442). As a result, six items have been included in addition to the eight items on the ATGS, in order to take into account issues that are more specifically related to circumstances in the Basque Country.

Thus, Table 4 presents the mean scores for each item used to assess attitudes toward gambling in Basque Country, based on a 4-point Likert scale ranging from 1 (Strongly Agree) to 4 (Strongly Disagree). These six additional items were designed to reflect specific social concerns and policy debates surrounding gambling in the Basque Country. The results show particularly high agreement with the item “Online gambling is more dangerous than face-to-face gambling” (M = 3.54), highlighting a strong perception of risk associated with digital gambling environments. Similarly, participants strongly agreed that “There should be more control over access to gambling” (M = 1.56) and that “Gambling advertising leads to more gambling” (M = 1.53), suggesting widespread support for regulatory measures.

The statement “Gambling is a means to generate income” (M = 2.53) reveals a moderately ambivalent view, hovering near the threshold for a positive attitude. Meanwhile, the item “Gambling has positive effects on the economy” (M = 2.43) reflects a slightly more favorable stance, albeit still below the positive-attitude cut-off. Taken together, these items underscore a generally critical view of gambling in the Basque Country, particularly in relation to its accessibility, advertising, and online formats.

**Table 4:** Items in the synthetic index (SIAG)

Items	Mean	Category of response
People should have the right to gamble whenever they want. †	1.83	1–4 (Strongly Agree-Strongly Disagree)
There are too many opportunities for gambling nowadays.*	1.50	
Gambling should be discouraged.*	1.66	
Most people who gamble do so sensibly.	2.66	
Gambling is dangerous for family life.*	1.45	
On balance gambling is good for society.	2.39	
Gambling livens up life.	2.57	
It would be better if gambling was banned altogether.*	2.54	
Gambling can be addictive*	1.49	
Gambling has positive effects on the economy	2.43	
Online gambling is more dangerous than face-to-face gambling.†	3.54	
Gambling is a means to generate income.	2.53	
There should be more control over access to the gambling,*	1.56	
Gambling advertising leads to more gambling.*	1.53	

*Items that were reversed

†Are not included in the index after factor analysis

*>*2.5 positive attitude towards gambling

Source: prepared by the authors based on the survey ‘Social perception and assessment of gambling in the Basque Country 2022’

The Principal Components technique enables a great deal of statistical information to be summarised in a few explanatory factors (Hair et al., 2019), making it a fundamental tool for understanding the underlying structure of attitudes. First, all the variables for which information was available were included. Second, due to the communalities of these variables and their explanatory power in the factor and model, two of the variables were excluded from the analysis^2^. Four factors were selected according to the eigenvalue greater than 1 method, which account for 66.2% of the variance.

Although the items used for this analysis were measured on a 4-point ordinal scale, the use of Principal Components Analysis (PCA) is considered appropriate when the sample is sufficiently large and item distributions approximate interval-level assumptions (Norman, 2010). Several methodological studies support the robustness of PCA and factor analysis with ordinal data, especially when there are four or more response categories and communalities exceed 0.5 (Bartholomew et al., 2002). In this study, the contribution of all variables included in the final solution was above 0.5, and both the Kaiser-Meyer-Olkin (KMO) measure of sampling adequacy (0.68) and Bartlett’s test of sphericity (*p* < 0.001) confirmed the suitability of the data for factor analysis. The resulting four-factor solution accounted for 66.2% of the total variance and showed adequate internal consistency, with a Cronbach’s alpha of 0.784. While the ordinal nature of the items is acknowledged as a limitation, the analysis yielded statistically robust and conceptually interpretable components that form the basis for the Synthetic Index of Attitudes toward Gambling (SIAG).

The matrix (Table 5) shows the main dimensions which in total and for each one in relation to its relative weight, will form the Synthetic Index of Attitudes towards Gambling (SIAG):

- Factor 1 - Regulation: made up of the variables ‘gambling should be discouraged’, ‘advertising about gambling is bad’ and ‘there should be more control’. This accounts for 32.2% of the variability.
- Factor 2 - Dangerousness: formed by the variables ‘gambling is dangerous for the family’, ‘gambling generates addiction’ and ‘There are too many opportunities to gamble’. This is the second-ranked factor for explanatory power, with 17.1%.
- Factor 3 - Economic: formed by the variables ‘gambling generates income’, ‘gambling is a type of entertainment and leisure’ and ‘gambling is positive for the economy’. This accounts for 8.5% of the variability.
- Factor 4 - Responsible gambling: formed by the variables ‘responsible gambling’, ‘prohibited gambling’ and ‘good for society’. This is the factor with the lowest explanatory power, 8.4%, albeit very similar to the previous one.

**Table 5:** Rotated component matrix

	Component			
	1	2	3	4
Gambling should be discouraged.	0.814			
Gambling advertising leads to more gambling.	0.791			
There should be more control over access to gambling	0.643			
Gambling is dangerous for family life.		0.873		
Gambling can be addictive.		0.859		
There are too many opportunities for gambling nowadays.		0.606		
Gambling is a means to generate income.			0.806	
Gambling livens up life.			0.699	
Gambling has positive effects on the economy.			0.693	
Most people who gamble do so sensibly.				0.869
It would be better if gambling was banned altogether.				0.709
On balance gambling is good for society.				0.546

Source: prepared by the authors using the SPSS software package

**Fig. 1:**
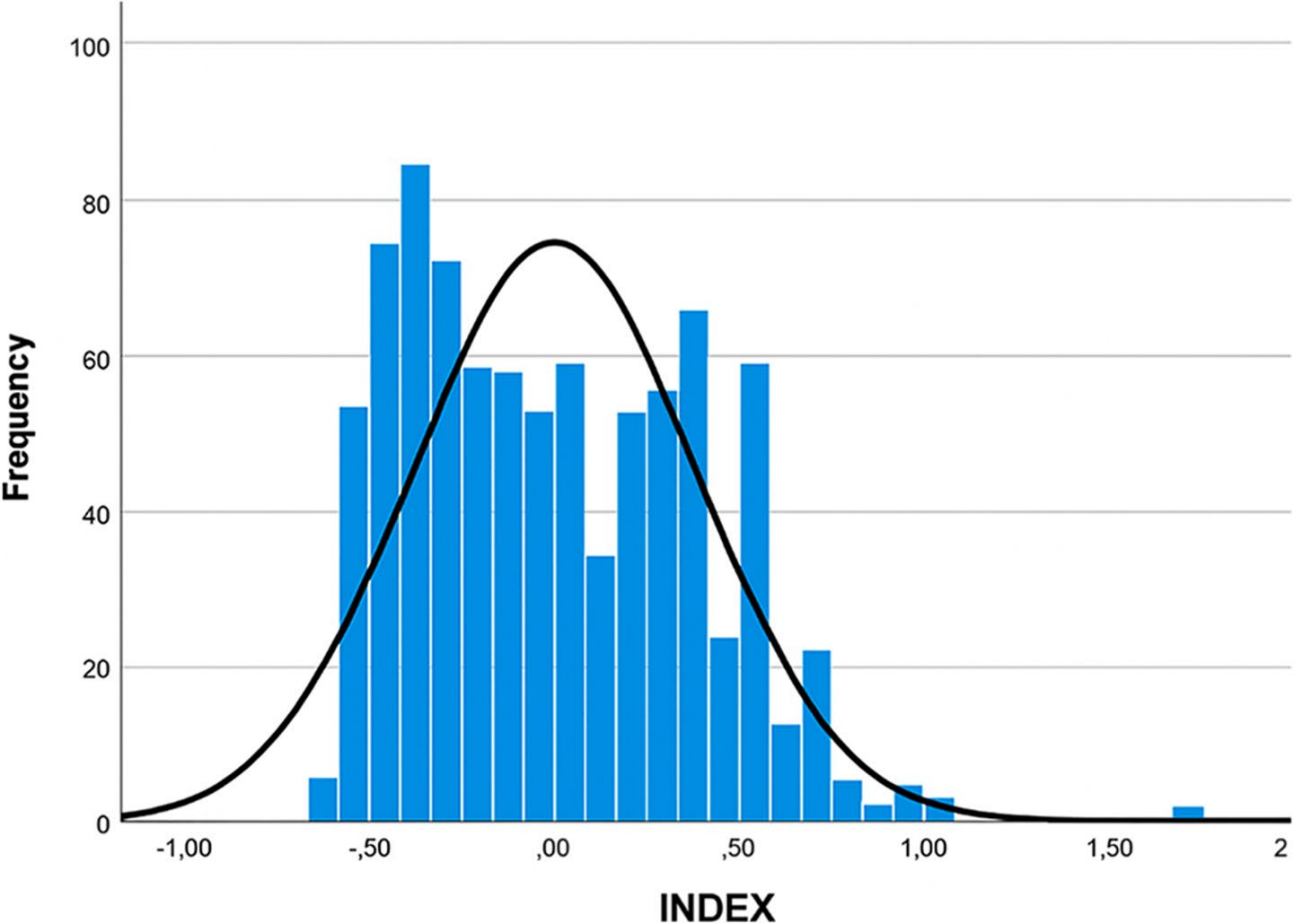
ISAG Distribution, Histogram. Source: prepared by the authors using the SPSS software package

These four dimensions were combined and weighted to create a synthetic index. The factor scores were saved as variables and standardised using the regression method. All things considered, the SIAG provides a simple and unique picture of the situation. An index ranging from − 0.60 to −1.69 was constructed as a result of the four dimensions identified in the principal component factor analysis, with a minimum score of −0.60, which would indicate a completely negative attitude to gambling, and a maximum value of −1.69, which on the contrary, would indicate a completely positive attitude (Fig. 1).

The inclusion of the new items resulted in a marked improvement in the psychometric performance of the scale. Cronbach’s alpha increased from 0.719 (ATGS-8) to 0.784 in the SIAG, indicating greater internal consistency. Similarly, the variance explained by the factorial analysis increased from 51.0 to 66.4%, suggesting a greater ability to account for the complexity of attitudes toward gambling.

Response patterns also reinforce the relevance of these additions. Participants strongly agreed with statements emphasizing the risks associated with gambling and supported stricter regulation of access and advertising. Although attitudes were somewhat more ambivalent about the potential economic benefits of gambling, the general orientation remained cautious and risk-conscious. These results confirm that adapting standardized instruments to specific sociocultural settings not only improves their reliability and explanatory power, but also provides a more accurate and context-sensitive view of public opinion, especially in settings where gambling is the subject of public opinion.

## Results of the Synthetic Index of Attitudes to Gaming (SIAG)

The analysis of the SIAG according to different socio-demographic and behavioural variables shows major differences in both the means obtained and in the levels of statistical significance (see Table 6). In terms of gender, women tend to have more negative attitudes towards gambling than men (mean = −0.0237 vs. 0.0244), although the difference does not reach the conventional threshold of statistical significance (*p* = 0.076).

Age is a variable with greater explanatory weight in attitudes towards gambling. Young people, especially those aged 18–29 years, show markedly negative attitudes (mean = −0.0512), in contrast to those aged 45–64 years (mean = 0.0075, *p* = 0.047) and more clearly, to those aged 65 years and older (mean = 0.0564, *p* = 0.003). The 30–44 age group also shows a negative attitude (mean = −0.0368), which differs significantly from the older age group (*p* = 0.006). These data point to a trend whereby attitudes towards gambling become more positive with age.

Educational level also influences opinions of gambling. People with higher education present more negative attitudes (mean = −0.0414) than those without higher education (mean = 0.0176), with a difference bordering on statistical significance (*p* = 0.053). This relationship suggests a more critical attitude to gambling among those with higher education.

Employment status also shows significant differences: students have a negative attitude towards gambling (mean = −0.063), while pensioners show a more positive attitude (mean = 0.0291), a statistically significant difference (*p* = 0.038). This pattern can be linked to both life experiences and exposure to gambling at different stages of the life cycle.

With regard to income level, the data show a significant association (*p* = 0.001) between monthly income and attitudes towards gambling. People with an income below €1,500 (mean = −0.083) and those with an income above €2,000 (mean = −0.1164) have more negative attitudes, while the group with an intermediate income (€1,501-2,000) has the most positive attitude (mean = 0.0839).

With regard to religion, no statistically significant differences were observed between believers (mean = 0.0085) and non-believers (mean = −0.0072), the p-value being 0.31. This variable therefore does not seem to play a major role in shaping attitudes towards gambling.

Finally, engaging in gambling and the frequency of doing so turn out to be decisive factors in the formation of attitudes. People who do not gamble present a clearly negative attitude (mean = −0.1087), while those who do gamble express a positive attitude (mean = 0.037), with a highly significant difference (*p* = 0.001). Furthermore, this relationship is accentuated when frequency is considered: sporadic players have a negative attitude (mean = −0.0405), while regular or habitual gamblers show a strongly positive attitude (mean = 0.1303), also with a high level of statistical significance (*p* = 0.001). These results suggest that frequent contact with gambling contributes to a more favourable opinion of it.

**Table 6:** SIAG related variables

Negative attitudes to gambling			Positive attitudes to gambling		
Mean		Variable	Sig		mean
−0.0237	Woman	Sex	0.076	Man	0.0244
−0.0512	18–29 years	Age	0.047	45–64 years	0.0075
−0.0512	18–29 years	Age	0.003	> 65	0.0564
−0.0368	30–44 years	Age	0.006	> 65	0.0564
−0.0414	High level	Educational attainment	0.053	Non high	0.0176
−0.063	Student	Employment situation	0.038	Pensioner	0.0291
−0.083	< 1,500€	Income	0.001	1,501–2000€	0.0839
−0.1164	> 2,001€	Income	0.001	1,501–2000€	0.0839
−0.0072	Non-religious	Religion	0.31	Religious	0.0085
−0.1087	No	Gamble	0.001	Yes	0.037
−0.0405	Sporadic	Gambling frequency	0.001	Regular	0.1303

Source: prepared by the authors using the SPSS software package

In order to obtain more information and to continue studying the variables related to attitudes, values and beliefs towards gambling that most discriminate among the Basque population, we applied a multivariate analysis of ‘K-Means’ classification. To that end, the Basque population was classified into three large groups: anti-gambling, ambivalent and pro-gambling. The criterion variables chosen to classify the Basque population into these three groups are the scores obtained in the four dimensions that are the basis for calculating the Index. These are: regulation, danger, economic and responsibility.

**Fig. 2:**
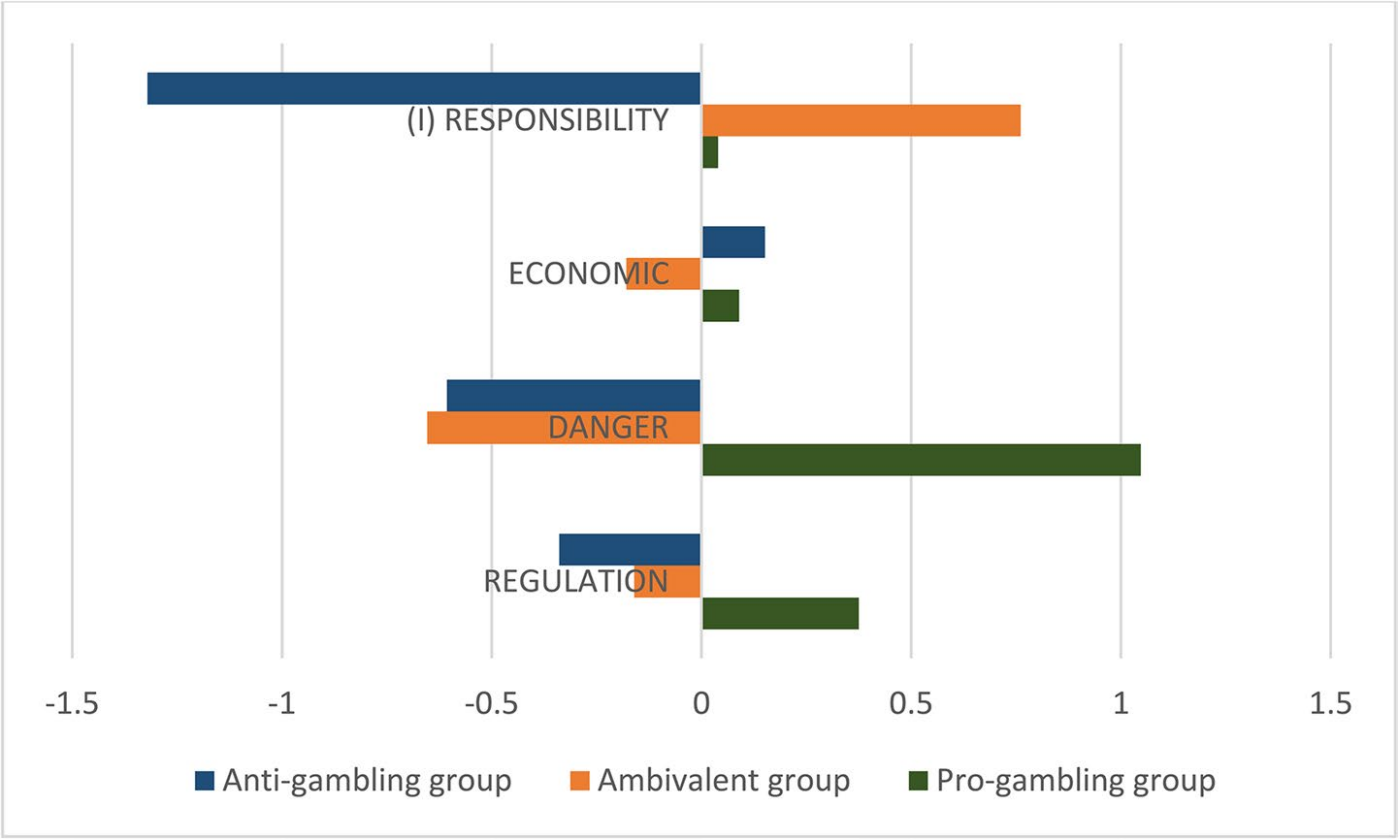
Mean scores for the groups. Source: prepared by the authors using the SPSS software package

Once each person was assigned to one of the three groups, depending on the answers given to the items and the scores obtained in the four dimensions, we analysed the behaviour and characteristics of those groups. Table 7 shows that the differences between the three groups are considerable. However, in order to facilitate understanding and interpretation thereof, these same scores are presented graphically (Fig. 2).

**Table 7:** Final cluster centres

	Anti-gambling group	Ambivalent group	Pro-gambling group
REGULATION	− 0.33913	− 0.16032	0.37491
DANGER	− 0.60709	− 0.65412	1.04755
ECONOMIC	0.15159	− 0.17916	0.08959
(I)RESPONSIBILITY	−1.32054	0.76077	0.03973

Source: prepared by the authors using the SPSS software package

The figure below shows that the group we categorised as ‘anti-gambling’ strongly agrees that gambling is dangerous, and with the need for control and regulation. To a certain extent, they believe that gambling can have an economic impact. However, the ‘pro-gambling’ group strongly disagrees with regulation, and with the idea that gambling is dangerous. Furthermore, this group does not consider a prohibition of gambling to be necessary, as most gamblers gamble responsibly and do create some positive economic impact. The final group, ‘ambivalent attitude towards gambling’, shows no clear pattern in their attitudes, although they tend towards negative positions towards gambling rather than positive ones: on the one hand, they do not consider that regulation is so necessary, although they do believe it is dangerous to some extent. This group is unclear about the economic contribution, but believes that the vast majority of gambling is responsible.

It can therefore be seen that the economic dimension presents the fewest differences between the three groups, with the mean being fairly central. However, the image of danger created by gambling polarises the ‘against’ and ‘in favour’ groups, as it is self-evident for the former and non-existent for the latter. Regulation and control of gambling are also very important issues for the anti-gamblers and not very important for the pro-gamblers, and this occupies a central position among the ambivalent group.

In the absence of a qualitative analysis, these results indicate that the argument related to gambling’s economic contribution does not seem to be a determining factor in attitudes to gambling, and yet the danger involved does polarise attitudes towards the extremes (in favour or opposed).

## Discussion

In this study, we found one of the most widely used scales, the ATGS, to be ineffective. The lack of significant results for this scale prevents us from making progress in the analysis of attitudes and population profiles, and as such we decided on an alternative index design through factor analysis, which as the results show, produces an interesting indicator. However, it is worth reflecting on and discussing the reasons or possible reasons why the ATGS scale is not appropriate for circumstances in the Basque Country.

The first issue is that there is little variability in the opinion of the population studied. The descriptive analysis shows a population in which 70% have a negative image of gambling, creating an asymmetrical distribution with little variability. The few intermediate and positive positions on the items on the scale make it necessary to include other items that include this variability, which is fundamental for the multivariate statistical analysis.

The second issue, which is in turn closely linked to the first, is cultural. Although the majority of Basque society has a negative image of gambling, the majority have also gambled in the last year, thereby creating some degree of dissonance. This is a reflection of societies with a Catholic origin and culture, in which gambling is considered ‘immoral’, but at the same time, sporting or festive celebrations are accompanied by traditions linked to gambling (the Christmas lottery, sports betting on major competitions and the Father’s Day draw are some examples of long-standing traditions). In fact, once again, the descriptive data show that people who say they are Catholics have a more positive image of gambling. It can therefore be stated that with its Catholic tradition, despite being relatively secularised, the Basque Country has a strong component of social desirability that we must attempt to overcome with the quantitative techniques used to construct the questionnaire, in order to adequately reflect the real scenario. Among other things, this led to the decision to reduce the scale of the items from five points to four, avoiding intermediate positions, also known as the refuge category, and forcing the respondent to adopt a position in favour or against. While helping to avoid social desirability, this forced polarisation into four positions produces even less statistical variability, partly contributing to the lack of significant results with certain scales.

Third, the debate on gambling has become politicised and part of the media and political agenda in recent years, after being heavily influenced by regulation and by advertising in particular (Morera Hernández, 2020). In short, left-leaning positions advocate regulating gambling to a greater extent and greatly limiting advertising (spaces, schedules), while positions leaning to the right are less interventionist (Buil et al., 2015). As a result, when attempting to measure attitudes towards gambling in the Basque context, it would be surprising if variables related to regulation and advertising were not included, forcing this index to incorporate new variables that are not included in the ATGS.

The fourth reason may be related to the modification of the scale, which entails a series of limitations that must be acknowledged in order to appropriately assess the scope and generalizability of the results. The main methodological limitation lies in the adaptation of the ATGS-8 response format, as a 4-point scale was used instead of the original 5-point scale that includes a midpoint. Although this decision is theoretically and empirically grounded—and has been extensively discussed in the methodology section—it prevents the direct application of the validated sum score (ranging from 8 to 40) and, consequently, limits comparability with other international studies that have strictly followed the original protocol of the scale (Wardle et al., 2011; Salonen et al., 2014).

The use of forced-choice scales (without a midpoint) has been supported in the literature when dealing with sensitive topics or contexts governed by strong social norms (Garland, 1991; Krosnick, 1991; Tourangeau et al., 2000). In situations where high social desirability is expected—that is, a tendency to provide socially acceptable rather than genuine responses—the midpoint may serve as an avoidance strategy or a “neutral escape” (Revilla & Saris, 2013). This risk is particularly relevant in the Basque Country, where gambling has historically been associated with negative values, especially among older generations, influenced by moral norms rooted in Catholic tradition and by a clearly preventive institutional discourse surrounding gambling. As documented by the Basque Gambling Observatory (Gobierno Vasco, 2017, 2022), the social image of gambling in Euskadi is significantly more negative than in other Spanish regions, and the rejection is widespread across age, gender, and social class. This reinforces the appropriateness of applying mechanisms to control normative bias.

Despite this contextual justification, it must be acknowledged that the choice of a 4-point scale is not neutral from a comparative standpoint. It not only alters the total possible range of scores but also affects the interpretation of mean values and the ability to classify respondents as “neutral”—a key element in many applications of the ATGS-8. The inability to calculate the validated sum score means that international thresholds (e.g., scores below 24 indicating negative attitudes) are not directly applicable. Therefore, the results should be understood as an internal snapshot of the Basque case rather than as a measure that can be compared in absolute terms. From a replicative logic, this methodological aspect may be perceived as a limitation, but it is also a strength in terms of internal validity, as it aligns with the specific features of the context.

Another limitation resulting from this adaptation is that, due to the absence of an explicit neutral option, respondents who were truly undecided may have felt compelled to take a position. This introduces a certain tension between external validity (comparability) and ecological validity (contextual fidelity). To mitigate this, a specific data collection protocol was implemented: interviewers were instructed not to offer a “don’t know/no answer” option, but to record it if the respondent explicitly requested it. This strategy aimed to avoid automatic neutrality while still allowing for the expression of genuine ambivalence.

Lastly, it is important to emphasize that the aim of this study was not to replicate the ATGS-8 internationally, but rather to understand gambling attitudes within a specific sociocultural context. This limitation, however, opens a valuable avenue for future research. It would be beneficial to explore ways of harmonizing adapted and original measures—e.g., through calibration studies or methodological experiments comparing forced-choice and midpoint-inclusive scales within the same population.

Finally, apart from the reflections and discussions derived from the scales and indices and the variables that form part of their construction, we believe that the quantitative debate should be contrasted with the qualitative debate. An index (the SIAG) has been developed within this initial attempt to measure and operationalise the attitude towards gambling. The SIAG has been contrasted with socio-demographic characteristics and subdivided into population groups (favourable, ambivalent and opposed). We believe that the logical next step, which would provide high quality information, would be the qualitative exploration of the discourses generated in each of these three groups.

## Conclusions

In conclusion, and briefly, this study aims to be an initial approach to the sociological study of gambling in the Basque Country. From a social point of view, it can not be denied that gambling is deeply rooted in society, both in terms of the amount of bets placed by the population, and the economic volume of the sector. However, as stated above, this does not mean that the image projected by gambling is consistent with this. Despite the fact that gambling takes place, it is still perceived as almost problematic or socially reprehensible behaviour. This is especially true of some forms of gambling.

For all these reasons, we have attempted to apply methodological approaches used in other contexts to the Basque case, but the results were not what we expected. As a result, the SIAG that has been developed in this text aims to be a tool for more in-depth study of the present situation in order to better understand the phenomenon and the prevailing attitudes towards gambling. Although it needs to be checked and validated in the future, it has provided some useful conclusions in this initial study, including the strong polarisation between groups and responses, except regarding gambling’s possible financial benefits.

This reinforces the position that this article has highlighted from the beginning, i.e. the idiosyncratic position of gambling in Basque public opinion. The pejorative attitudes towards gambling are not strong enough to reduce it, or to rule it out as a driving force in the economy.

## Footnotes

1 The Observatorio Vasco del Juego (the Basque Gambling Observatory): https://www.euskadi.eus/gobierno-vasco/observatorio-vasco-juego/.

2 The ‘People should have the right to gamble whenever they want’ and ‘Online gambling is more dangerous than face-to-face gambling’ variables were therefore eliminated because of their low contribution (0.25 and 0.45 respectively).
